# Characterization of the far-red light absorbing light-harvesting chlorophyll *a*/*b* binding complex, a derivative of the distinctive Lhca gene family in green algae

**DOI:** 10.3389/fpls.2024.1409116

**Published:** 2024-06-10

**Authors:** Makiko Kosugi, Shuji Ohtani, Kojiro Hara, Atsushi Toyoda, Hiroyo Nishide, Shin-Ichiro Ozawa, Yuichiro Takahashi, Yasuhiro Kashino, Sakae Kudoh, Hiroyuki Koike, Jun Minagawa

**Affiliations:** ^1^ Division of Environmental Photobiology, National Institute for Basic Biology, Okazaki, Japan; ^2^ Faculty of Education, Shimane University, Matsue, Japan; ^3^ Faculty of Bioresource Sciences, Akita Prefectural University, Akita, Japan; ^4^ Comparative Genomics Laboratory, National Institute of Genetics, Mishima, Japan; ^5^ Data Integration and Analysis Facility, National Institute for Basic Biology, National Institutes of Natural Science, Okazaki, Japan; ^6^ Institute of Plant Science and Resources, Okayama University, Kurashiki, Japan; ^7^ Research Institute for Interdisciplinary Science, Okayama University, Okayama, Japan; ^8^ Graduate School of Science, University of Hyogo, Ako-gun, Japan; ^9^ National Institute of Polar Research, Research Organization of Information and Systems, Tokyo, Japan; ^10^ Department of Polar Science, School of Multidisciplinary Science, The Graduate University for Advanced Studies, SOKENDAI, Tokyo, Japan; ^11^ Department of Biological Sciences, Faculty of Science and Engineering, Chuo University, Tokyo, Japan; ^12^ Graduate Institute for Advanced Studies, SOKENDAI, Okazaki, Japan

**Keywords:** photosynthesis, far-red (FR) light, Antarctica, light harvesting complex, draft genome assembly and annotation, RNA-seq, green algae, phylogenetic analysis

## Abstract

*Prasiola crispa*, an aerial green alga, exhibits remarkable adaptability to the extreme conditions of Antarctica by forming layered colonies capable of utilizing far-red light for photosynthesis. Despite a recent report on the structure of *P. crispa*’s unique light-harvesting chlorophyll (Chl)-binding protein complex (Pc-frLHC), which facilitates far-red light absorption and uphill excitation energy transfer to photosystem II, the specific genes encoding the subunits of Pc-frLHC have not yet been identified. Here, we report a draft genome sequence of *P. crispa* strain 4113, originally isolated from soil samples on Ongul Island, Antarctica. We obtained a 92 Mbp sequence distributed in 1,045 scaffolds comprising 10,244 genes, reflecting 87.1% of the core eukaryotic gene set. Notably, 26 genes associated with the light-harvesting Chl *a*/*b* binding complex (LHC) were identified, including four Pc-frLHC genes, with similarity to a noncanonical Lhca gene with four transmembrane helices, such as Ot_Lhca6 in *Ostreococcus tauri* and Cr_LHCA2 in *Chlamydomonas reinhardtii*. A comparative analysis revealed that Pc-frLHC shares homology with certain Lhca genes found in *Coccomyxa* and *Trebouxia* species. This similarity indicates that Pc-frLHC has evolved from an ancestral Lhca gene with four transmembrane helices and branched out within the Trebouxiaceae family. Furthermore, RNA-seq analysis conducted during the initiation of Pc-frLHC gene induction under red light illumination indicated that Pc-frLHC genes were induced independently from other genes associated with photosystems or LHCs. Instead, the genes of transcription factors, helicases, chaperones, heat shock proteins, and components of blue light receptors were identified to coexpress with Pc-frLHC. Those kinds of information could provide insights into the expression mechanisms of Pc-frLHC and its evolutional development.

## 1 Introduction

The light-harvesting antennae of photosystems play essential roles in the adaptation of photoautotrophs to the different light conditions on Earth (Green and Parson, 2003; Mirkovic et al., 2017). Light-harvesting chlorophyll (Chl) binding protein complexes (LHCs) form a large gene family in eukaryotic photoautotrophs (Dolganov et al., 1995; Koziol et al., 2007). All genes encoding LHCs are coded in the nuclear genome, not in the chloroplast genome. *Chlamydomonas reinhardtii*, a model green alga, has 9 and 11 LHCI (*LHCA1-9*) and LHCII (*LhcbM1-9, LHCB4, LHCB6*) genes, respectively, whose function and structural aspects have been extensively studied (Elrad et al., 2002; Stauber et al., 2003; Minagawa and Takahashi, 2004; Tokutsu et al., 2004). LHCI and LHCII proteins in plants and green algae act as peripheral light-harvesting antennae for photosystems I and II (PSI and PSII), respectively, which conduct charge separation and drive electron transfer. LHCII proteins function as trimers or monomers in the PSII complex: Major LHCII polypeptides act as LHCII trimers (LhcbM1-9) and CP26 and CP29 act as minor monomeric LHCII (Burton-Smith et al., 2019; Shen et al., 2019; Sheng et al., 2019). These LHCIIs surround the PSII core as peripheral light-harvesting antennae while LHCIs are bound to specific locations of the photosystem I core (Kubota-Kawai et al., 2019, Su et al., 2019; Suga et al., 2019). LHCA1and LHCA3-8 in *C. reinhardtii* form two layers of a crescent-shaped complex at the side of PSAF and PSAJ of the PSI core. The other two, LHCA2 and LHCA9, are bound to the side, between PSAG and PSAH, as heterodimers (Ozawa et al., 2018). All these LHCs have three transmembrane helices, except LHCA2, which has a fourth transmembrane helix (Su et al., 2019; Suga et al., 2019).

In general, LHC monomers bind 11–16 chlorophylls (Chl) and 3–5 carotenoids (Green and Parson, 2003). Chl *a*, the most abundant Chl in oxygenic photosynthetic organisms, exhibits a Soret band at approximately 435 nm and Qx and Qy bands at approximately 600–700 nm in organic solvents. The LHC proteins of Chlorophyta, which carry Chl *b* as well as Chl *a*, extend the absorption peaks to wavelengths >435 nm and <660 nm. The pigment–pigment and/or pigment–protein interactions also affect the absorption properties of LHCs (Mirkovic et al., 2017). Notably, the π-π interaction between the two porphyrin rings of Chls causes a large red shift in the Qy absorption band. This Chl coupling strategy is used in algae and plants for expansion of absorption spectra to wavelengths >700 nm, since they have no genes for synthesizing far-red absorbing Chls such as the Chl *d* or Chl *f* found in some cyanobacteria (Miyashita et al., 1996; Chen et al., 2012). The PSI core complex in cyanobacteria and the PSI–LHCI supercomplex in algae typically have coupled Chl*a*s called “red Chls” to extend the absorption band to the far-red region (Croce and van Amerongen, 2020). However, only a few red Chls were generally found in PSII or LHCII in normal plants (Pettai et al., 2005; Laisk et al., 2014). This is probably because of the larger energy gap between red Chls and those in the reaction center of the PSII (P680). When the energy absorbed by red Chls is used to excite P680, a huge uphill excitation energy transfer (uphill EET) is required, although it is not easily accomplished. Nevertheless, remarkable far-red absorption bands have been reported in two green algae, *Ostreobium*.sp (Koehne et al., 1999; Wilhelm and Jakob, 2006) and *P. crispa* (Kosugi et al., 2020), and four stramenopiles, *Phaeodactylum tricornutum* (Fujita and Ohki, 2004), *Chromera velia* (Bína et al., 2014; Kotabová et al., 2014), *Trachydiscus minutus* (Bína et al., 2019) and *Eustigmatophyceae* sp. FP5 (Wolf et al., 2018), as follows. A green alga *Ostreobium* sp., which lives in coral reefs under extreme low-light conditions, has a 22 kDa LHC binding red Chl *a* and shows discernible absorbance shoulder at approximately 710 nm (Koehne et al., 1999; Wilhelm and Jakob, 2006). *P. tricornutum* (a diatom) expresses a red Chl *a* -binding LHC that shows an absorbance peak at 703 nm in addition to a peak at 680 nm when grown under red light conditions (Fujita and Ohki, 2004). *C. velia* (Alveolata) shows a red-shifted absorbance with a maximum around 705 nm when cultured under red light, and a 17 kDa fucoxanthin-chlorophyll *a*/*c* protein complex-like protein that binds red Chl *a* (Bína et al., 2014; Kotabová et al., 2014). *T. minutus* (Xanthophyceae) has a red–violaxanthin–Chl *a* protein (rVCP) that binds violaxanthin (vio), non-esterified vaucheriaxanthin, and Chl *a* (Bína et al., 2019). It has been proposed that one of the low-energy Chl *a* molecules in rVCP absorbs light at ≥700 nm. *Eustigmatophyceae* sp. FP5 shows a large absorption peak at 705 nm when cultured under far-red light conditions, and the expression of several far-red-absorbing LHCs that bind red-shifted Chl *a* was confirmed by clear native PAGE (Wolf et al., 2018).

*P. crispa*, an aerial green alga, is one of the dominant algae in the terrestrial ice-free area of Antarctica (Broady, 1979, 1996). Sheet-like thallus of *P. crispa* form layered colonies. The surface of such colonies faces a severe environment with strong sunlight, including UV radiation (Kosugi et al., 2018). However, while the cells in the inner layer of the colony suffer less photodamage, oxygenic photosynthetic activity is largely limited by the low visible light environment. Infrared (>700 nm) is the most abundant radiation under such conditions (Kosugi et al., 2023). Recently, *P. crispa* was found to exhibit a remarkable absorption shoulder around 706 nm, and a unique far-red light-absorbing LHC, Pc-frLHC, was identified. PSII excitation with far-red light energy absorbed in the Pc-frLHC can be achieved with as high efficiency as visible light by measuring the action spectrum of photosynthetic oxygen evolving activity (Kosugi et al., 2020). Previously, the total amino acid sequence of Pc-frLHC was estimated from transcriptome data (PRJNA329112) using three partial internal amino acid sequences of the purified Pc-frLHC of *P. crispa* harvested from the Soya Coast of East Antarctica. One potential protein wherein the three partial amino acid sequences completely matched was selected (UniProt ID: C0HLU5). As such, we constructed the structural model of Pc-frLHC as an undecameric 11-fold symmetric complex at 3.13 Å resolution using the deduced amino acid sequence and the electron density map produced with cryo-EM (Kosugi et al., 2023). Each LHC subunit has four transmembrane helices and binds 11 Chls and two carotenoids (loroxanthine and vio). From the calculation of exciton coupling, the unique trimeric Chls, Chl603-609-708, were assigned to far-red absorbing Chls (Kosugi et al., 2023).

However, the nuclear genomic sequence of *P. crispa* including LHC genes has not been reported so far. These data are essential for the molecular analysis of Pc-frLHC and to clarify the uphill excitation energy transfer mechanism and its evolution. In this study, we drafted the genome sequence of a cultured strain, *P. crispa* 4133 and performed a phylogenetic analysis of LHC candidates in *P. crispa* 4133 to reveal the evolutionary process of the unique LHC complex.

## 2 Materials and methods

### 2.1 Antarctic soil sampling

Syowa Station is located in the northern part of East Ongul Island (69°00’S, 39°35’E) in Lützow-Holm Bay, East Antarctica. In the austral summer, most of the island is snow-free and a sandy soil is exposed. Surface soil was collected near a sewage outfall after secondary treatment in February 2000 by members of the 41st Japanese Antarctic Research Expedition. No *P. crispa* colonies could be observed with the naked eye. The sample was collected using a sterilized spoon, placed in a sterilized plastic bottle, and brought back to Japan. The samples were stored at −20°C.

### 2.2 Algal strain and growth conditions

A 0.5 g soil sample, melted at room temperature, was inoculated onto a Petri dish containing 1.5% agar Bold’s Basal Medium (BBM) (Bischoff and Bold, 1963) on a clean bench. Cells were grown under standard growth conditions of illumination at 6.4–20 μmol photons m^−2^ s^−1^ using a cool-white-fluorescent lamp with a 12-h light and 12-h dark cycle at 15°C. After two months of incubation, green filamentous colonies of *P. crispa* were observed on the agar surface. They were picked up with a sterilized needle and transferred to a new plate containing BBM, 1.5% agar, and 10 ppm of benomyl, which prevents fungi growth. After confirming that it developed into a monoalgal culture strain under a microscope (Olympus, BX60), strain 4113 was stored on a 1.5% agar slant of BBM. For genome sequencing, we prepared the culture strain 4113_Pc2 isolated from one filament of the strain 4113 to make the genome sequence uniform in 2017. The strain was registered on the BioSample of DDBJ as SAMD00649041.

### 2.3 Growth conditions to induce Pc-frLHC accumulation for transcriptome analysis and biochemical characterization

Pc-frLHC expression in the cultured strain 4113 is induced under red LED light but suppressed under white fluorescent light. *P. crispa* strain 4113 cells, cultured on agar plates containing BBM, 0.05% peptone, and 0.1% glucose, were grown under fluorescent light for two months. To induce Pc-frLHC, the samples were illuminated at 30 μmol photons m^−2^ s^−1^ using a red LED, whose spectrum is shown in **Supplementary Figure 1**. For transcriptome analysis, *P. crispa* strain 4113 cells cultured under fluorescent light for 2 months were transferred to the condition under the red LED light and incubated for 6 or 12 days. Harvested cells were placed into sample tubes, immediately frozen in liquid nitrogen, transferred to Akita Prefectural University, and maintained <70°C.

### 2.4 Thylakoid membrane isolation and subsequent electrophoresis analysis

Thylakoid membranes were prepared from *P. crispa* 4113 cells as described previously (Kosugi et al., 2023). Absorption spectra were measured using an MPS-2450 spectrometer (Shimadzu, Kyoto, Japan) with a slit width set to 2.0 nm. The spectra were normalized by setting the absorbance at the peak of the Qy band to 1. The absorbance at 750 nm was set to baseline.

High-resolution clear native (hrCN)-polyacrylamide gel electrophoresis (PAGE) was performed using a stacking gel containing 2.91% acrylamide (A.A.) and 0.09% bis acrylamide (bis A.A.) and a separation gel comprising a linear gradient of 3.84%–12.48% (w/v) of A.A., 0.12%–0.39% (w/v) bis A.A., and 0%–15.5% (w/v) glycerol. Both gels contained 25 mM Imidazole-HCl and 0.5 M 6-aminocaproic acid. The gel was pre-run with cathode buffer (25 mM imidazole/HCl, pH 7.0) and anode buffer (0.03% n-Dodecyl-β-D-maltoside (β-DDM), 50 mM Tricine, 7.5 mM imidazole, pH 7.0) with a constant current of 4 mA at 4°C for 30 min. The thylakoid sample was solubilized with 1% (w/v) β-DDM at a Chl concentration of 0.5 mg Chl/mL for 20 min on ice and centrifuged at 20,000 × g at 4°C for 10 min. Then, the supernatant was applied to the gel. Electrophoresis was first performed with a constant current of 2~4 mA during which samples pass through a stacking gel and then with a constant voltage of 100 V at 4°C. Blue native (BN)-PAGE was performed with the same condition to hrCN-PAGE except for that 0.02% CBB G-250 added into the anode buffer and the concentration was reduced to 0.002% after samples pass through a stacking gel. After electrophoresis, each BN-PAGE lane was cut and incubated in 60 mM dithiothreitol (DTT), 4% sodium lauryl sulfate (SDS), 60 mM Tris-HCl (pH 8.6), 7.5 M urea, and 1% bromophenol blue solution to denature and solubilize separated protein complexes in the gels, and used for 2D-SDS-PAGE analysis.

2D-SDS-PAGE was performed with a stacking gel containing 6% (w/v) A.A. and 0.08% (w/v) bis A.A. and a separation gel comprising 15% (w/v) A.A and 0.39% (w/v) bis A.A. Both gels contained 7.5 M urea, 0.375 M Tris-HCl (pH 8.4), and 0.1% (w/v) SDS. hrCN-PAGE gels incubated in the DTT solution were placed on stacking gels and fixed with 1% agarose. Electrophoresis was performed with a constant current of 9–22 mA. Separated proteins in SDS-PAGE gels were silver-stained.

### 2.5 RNA extraction, cDNA library construction, and RNA sequencing

For each light condition, five separate culture plates were prepared. These conditions included white fluorescent light and either 6 or 12 days under red LED light. Cells harvested from one plate were placed in a plastic tube and immediately frozen in liquid nitrogen. Frozen samples were ground to powder using a Multi-beads Shocker (Yasui Kikai Corporation, Osaka, Japan). Total RNA was extracted using the QIAGEN RNeasy Plant Mini Kit (Qiagen). Contaminating DNA was eliminated by DNase I treatment. The characteristics of each sample are shown in **Supplementary Table 1**. Samples were named according to the following rule: The first and second numbers of the sample name indicate the irradiation period (days) and the culture plate number, respectively. Two high-quality samples were selected from three samples under each light condition. Total RNA samples of 0–1, 0–3, 6–2, 6–3, 12–2, and 12–3 were used for Illumina cDNA library preparation. The cDNA library was constructed using a TruSeq RNA Sample Prep Kit ver.2 (Illumina) at the Biotechnology center of Akita Prefectural University and sequenced on a HiSeq 2500 sequencer (Illumina) at Macrogen Japan Corp (Tokyo, Japan). Raw reads generated from six samples were adapter-trimmed using FASTX-Toolkit (ver. 0.0.14, fastq_quality_filter), Prinseq (ver. 0.20.4), and Trimmomatic (ver. 0.36). Read quality was assessed using FastQC (ver. 0.11.3). Of 539,221,328 raw reads, 225,045,386 read pairs were acquired. After quality control, reads were used for gene prediction in the draft genome sequence of *P. crispa* and differential expression gene (DEG) analysis.

### 2.6 Genome sequencing and draft genome assembly

Whole-genome sequencing was performed using PacBio and Illumina sequencing platforms. Genomic DNA was isolated from *P. crispa* using the CTAB method and a Genomic-tip Kit (QIAGEN, Hilden, Germany). A continuous long read (CLR) SMRTbell library was prepared using the SMRTbell Express Template Prep Kit (Pacific Bioscience, CA, USA) according to manufacturer’s instructions. The CLR library was size-selected using the BluePippin system (Saga Science, MA, USA) with a lower cutoff of 30 kb and sequenced on 10 SMRT cells (1M v3 LR) using the Sequel system and Binding Kit 3.0/Sequencing Kit 3.0 (Pacific Bioscience, CA, USA), with 1,200 min movies. Next, paired-end and 15-kb mate-pair libraries were constructed using a TruSeq DNA PCR-Free Library Prep Kit (Illumina, CA, USA) and a Nextera Mate-Pair Sample Preparation Kit (Illumina, CA, USA), respectively. Two sequencing libraries were sequenced on the Illumina HiSeq 2500 system with 2,250 bp reads for the paired-end library and 2,100 bp reads for the mate-pair library.

The assembly process was initiated by mapping PacBio subheads to the organellar genome via minimap2 (v2.11-r797) to selectively exclude organellar reads. The “-x map-pb” parameter was used to accommodate the unique characteristics of PacBio subheads. Subsequently, filtered subheads were assembled using Flye (v2.4.2) with the parameter “-g 100m,” thereby generating an initial assembly. The initial assembly was then subjected to several iterative correction steps. First, 15-kb mate- pair reads were mapped to the initial assembly using SMALT (v0.7.6) with parameters “map -x -r 1-i 20,000-j 0.” Misassemblies were identified using the mapped 15-kb mate-pair reads and subsequently corrected with REAPR (v1.0.18), resulting in an updated assembly. Next, the updated assembly was subjected to another correction step, involving mapping of 600-bp paired-end reads using BWA (v0.7.12) with the parameter “mem.” Using the mapping results, subsequent nucleotide-level corrections were implemented using Pilon (v1.22), to produce a refined assembly. In the final verification step, the refined assembly was mapped to the RefSeq bacterial genome database using blastn (v2.2.9) with an E-value cutoff parameter of “-e 1e-30’ to identify and eliminate any contaminants, resulting in the final assembly. The genome sequence data was registered on the DDBJ Sequence Read Archive (DRA) under accession numbers DRA017229. The accession number of BioProject is PRJDB15914.

### 2.7 Repeat masking

To mask the *P. crispa* draft genome, a custom repeat library was constructed using RepeatModeler (v.2.0.3) (Flynn et al., 2020). The genome was masked using RepeatMasker (v.4.1.2) and the repeat library generated with RepeatModeler.

### 2.8 Gene prediction

Gene prediction was performed on the soft-masked *P. crispa* draft genome sequence using BRAKER2 in two distinct runs (Brůna et al., 2021). In the first run, BRAKER2 used hints derived from RNA-seq mapping results. RNA-seq reads were aligned to the hard-masked genome using HISAT2 with default parameters (Kim et al., 2019). The resulting alignment file was then provided to BRAKER2 as a hint. For the second run, BRAKER2 was provided with protein hints derived from two sources: the “plants_protein” dataset from OrthoDB (Zdobnov et al., 2021) and the amino acid sequences from *C. reinhardtii*. These protein sequences were aligned to the soft-masked genome, which subsequently served as hints for BRAKER2 gene prediction. In either run, prediction was performed on the soft-masked genome. The predictions obtained from both BRAKER2 runs were merged using TSEBRA (Gabriel et al., 2021). The configuration was modified with the “intron_support” parameter set to 0.2 according to authors’ recommendation when the default parameter was considered too strict (https://github.com/Gaius-Augustus/TSEBRA). To assess gene prediction completeness, Benchmarking Universal Single-Copy Orthologs (BUSCO) (v5; Manni et al., 2021) was performed in the protein mode. For BUSCO lineage datasets, eukaryota_odb10 was used. Gene annotation was performed using Blast2GO. Genes were assigned by number and described as “pcri_g + number.” The transcripts of each gene were described as “pcri_g + number+ t + number.” Proteins encoded by each transcript are described as “pcri_g + number+ t + number + p + number” (**Table 1**).

**Table 1:** The list of estimated LHC genes and the deduced transcripts and proteins in *P. crispa*.

	Gene ID	Transcript ID	Protein ID	Annotation	Abrrabiated names of Gene/protein
1	pcri_g8716	pcri_g8716.t1	pcri_g8716.t1.p1	Pc_frLHC	*Pc_frLHC1*/Pc_frLHC1
2	pcri_g8718	pcri_g8718.t1	pcri_g8718.t1.p1	Pc_frLHC	*Pc_frLHC2*/Pc_frLHC2
3	pcri_g8720	pcri_g8720.t1	pcri_g8720.t1.p1	Pc_frLHC	*Pc_frLHC3*/Pc_frLHC3
4	pcri_g8045	pcri_g8045.t1	pcri_g8045.t1.p1	Pc_frLHC	*Pc_frLHC4*/Pc_frLHC4
5	pcri_g1403	pcri_g1403.t1	pcri_g1403.t1.p1	Synonym of Cr_LHCA1	*Pc_LHCA1*/Pc_LHCA1
6	pcri_g5451	pcri_g5451.t1	pcri_g5451.t1.p1	Synonym of Cr_LHCA2	*Pc_LHCA2*/Pc_LHCA2
7	pcri_g7144	pcri_g7144.t1 pcri_g7144.t2	pcri_g7144.t1.p1 pcri_g7144.t2.p1	Synonym of Cr_LHCA3	*Pc_LHCA3*/Pc_LHCA3
8	pcri_g794	pcri_g794.t1	pcri_g794.t1.p1	Synonym of Cr_LHCA4	*Pc_LHCA4*/Pc_LHCA4
9	pcri_g2846	pcri_g2846.t1	pcri_g2846.t1.p1	Synonym of Cr_LHCA5	*Pc_LHCA5*/Pc_LHCA5
10	pcri_g795	pcri_g795.t1	pcri_g795.t1.p1	Synonym of Cr_LHCA6	*Pc_LHCA6*/Pc_LHCA6
11	pcri_g1402	pcri_g1402.t1	pcri_g1402.t1.p1	Synonym of Cr_LHCA7	*Pc_LHCA7*/Pc_LHCA7
12	pcri_g1401	pcri_g1401.t1	pcri_g1401.t1.p1	Synonym of Cr_LHCA8	*Pc_LHCA8*/Pc_LHCA8
13	pcri_g3642	pcri_g3642.t1	pcri_g3642.t1.p1	Synonym of Cr_LHCA9	*Pc_LHCA9*/Pc_LHCA9
14	pcri_g8360	pcri_g8360.t1	pcri_g8360.t1.p1	CP26	*Pc_PC26*/Pc_PC26
15	pcri_g2949	pcri_g2949.t1	pcri_g2949.t1.p1	CP29	*Pc_PC29*/Pc_PC29
16	pcri_g743	pcri_g743.t1	pcri_g743.t1.p1	LHCII	*Pc_Lhcb1*/Pc_Lhcb1
17	pcri_g2229	pcri_g2229.t1	pcri_g2229.t1.p1	LHCII	*Pc_Lhcb2*/Pc_Lhcb2
18	pcri_g2502	pcri_g2502.t1	pcri_g2502.t1.p1	LHCII	*Pc_Lhcb3*/Pc_Lhcb3
19	pcri_g3381	pcri_g3381.t1	pcri_g3381.t1.p1	LHCII	*Pc_Lhcb4*/Pc_Lhcb4
20	pcri_g5581	pcri_g5581.t1	pcri_g5581.t1.p1	LHCII	*Pc_Lhcb5*/Pc_Lhcb5
21	pcri_g5764	pcri_g5764.t1	pcri_g5764.t1.p1	LHCII	*Pc_Lhcb6*/Pc_Lhcb6
22	pcri_g8878	pcri_g8878.t1	pcri_g8878.t1.p1	LHCII	*Pc_Lhcb7*/Pc_Lhcb7
23	pcri_g9148	pcri_g9148.t1	pcri_g9148.t1.p1	LHCII	*Pc_Lhcb8*/Pc_Lhcb8
24	pcri_g1166	pcri_g1166.t1	pcri_g1166.t1.p1	LHCSR or HLIP or ELIP	
25	pcri_g3292	pcri_g3292.t1	pcri_g3292.t1.p1	HLIP or ELIP	
26	pcri_g7307	pcri_g7307.t1	pcri_g7307.t1.p1	LHCSR or HLIP or ELIP	

Gene prediction of organelle genomes was performed using Glimmer 2.0 packaged in an Omix Box (BioBam Bioinformatics Version 2.1.14).

### 2.9 RNA-seq analysis

HISAT2, StringTie, and edgeR (ver. 3.34.1) were used for transcript-level expression analysis of RNA-seq experiments. Sequenced reads whose quality was assessed using FastQC for each sample were aligned using HISAT2 by mapping to the *P. crispa* reference genome. hisat2-build created the index of the reference sequence before alignment. Each gene within the alignment was counted using StringTie. Count data were normalized for differential expression (DE) analysis with the TMM method by using calcNormFactors of edgeR. The DE test was performed between days 0 and 6 (DE_0-6), and between days 6 and 12 (DE_6-12), respectively. The log(FC), where FC stands for fold change of expression, and false discovery rate (FDR) values were used to identify genes whose expression was correlated with Pc-frLHC gene expression. Heatmaps were drawn by using a package, pheatmap, from R software. The values of the matrix are normalized by columns. The transcriptome sequence data was registered on the DRA under accession numbers DRA018642. The accession number of BioProject is PRJDB18065.

### 2.10 Mass spectrometry

The polypeptides separated by SDS-PAGE were visualized by silver staining and subjected to in-gel tryptic digestion for mass spectrometric (MS) analysis as described in Ozawa et al. (2009) with some modifications. Trypsin-digested peptides were subjected to LC-MS/MS (LTQ, Thermofisher scientific, USA), and the acquired mass spectral data were analyzed with Proteome Discoverer ver.2.1.1.21 (Thermofisher Scientific, USA) against the protein database constructed in this study. Post-translational modifications were not considered; a false discovery rate of 0.01 was calculated by the Decoy database search and used for all analyses.

### 2.11 Acquisition of LHC sequences of green algae and plants

As shown in **Supplementary Data 1**, phylogenetic analysis was performed with 27 deduced protein sequences estimated from 26 potential LHC genes of *P. crispa* (**Table 1**) and 66 reference protein sequences selected from the database, National Center for Biotechnology Information. LHC sequences of *C. reinhardtii*, *D. salina*, *B. corticulans*, *Ostreococcus tauri*, *Arabidopsis thaliana*, and *Cyanidioschyzon merolae* were used for phylogenetic analysis as their function and structural information in the photosystem and LHC supercomplexes have been well described. In addition, some nonannotated LHCs from other green algae showing high homology to Pc-frLHC were included in the dataset. The amino acid sequences of LHCs used for phylogenetic analysis are summarized in **Supplementary Data 1**.

### 2.12 Phylogenetic analysis of Pc-frLHC

The evolutionary history was inferred by using the maximum likelihood method and the JTT matrix-based model (Jones et al., 1992). We used the tree with the highest log likelihood (-29433.39). The percentage of trees wherein the associated taxa clustered together is shown next to the branches. Initial tree(s) for the heuristic search were obtained automatically by applying neighbor-joining and BioNJ algorithms to a matrix of pairwise distances estimated using the JTT model. Topologies with superior log likelihood values were then selected. A distinct Gamma distribution was used to model evolutionary rate differences among sites (3 categories [+G, parameter = 3.4208]). The tree was then drawn to scale, with branch lengths measured as the number of substitutions per site. A total of 75 amino acid sequences were analyzed to construct the tree. There were 547 positions in the final dataset. Evolutionary analyses were conducted using MEGA11 as reported by Tamura et al. (2021).

## 3 Results

### 3.1 Morphology of *Prasiola crispa*

**Figure 1:**
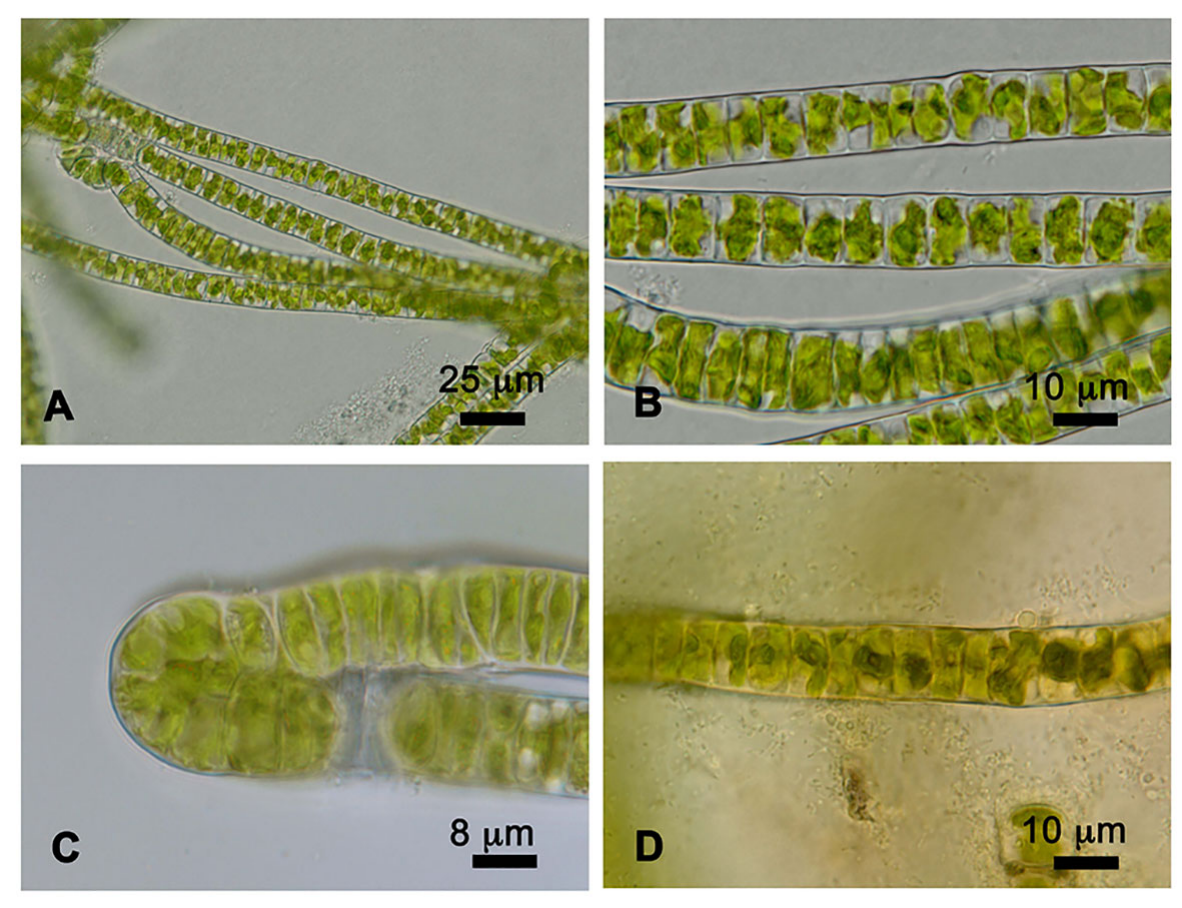
Cells of *Prasiola crispa* strain 4113. **(A, B)** One row of filaments. **(C)** Two rows of filaments. **(D)** Pyrenoids in the center of each cell stained with iodine solution.

*Prasiola crispa* is an ornithocoprophilous alga that forms macroscopic thallus colonies around the nesting sites of penguin rookeries and snow petrels in Antarctica. Under the culture conditions used in this study, strain 4113 formed single (**Figures 1A, B**) or double (**Figure 1C**) row filaments. Such filaments are straight and parallel to each other. Cells are quadrangular in shape, shorter in longitude than width: 11–15 μm in width, and 3.5–8 μm in length. Each cell contains a single, star-shaped chloroplast with a central pyrenoid (**Figure 1D**).

### 3.2 Observation of Pc-frLHC accumulation by red illumination

**Figure 2:**
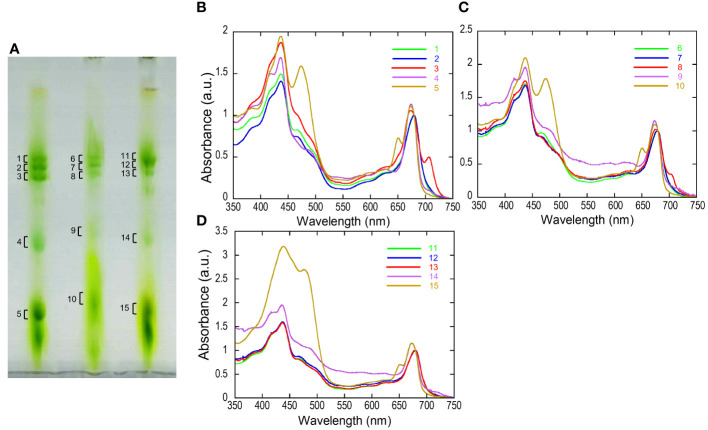
High-resolution clear native PAGE (hrCN-PAGE) for separation of photosystem complexes **(A)** and absorbance spectra of each green band after hrCN-PAGE **(B–D)**. Clear native PAGE of thylakoids isolated from *P. crispa* thali harvested from Antarctica (A, left), cultured strain incubated under red LED light (A, center), and cultured strain incubated under fluorescence light (A, right). The contrast of CN-PAGE photograph was increased for the ease of recognition of green bands. Panels B–D show the absorbance spectra of the green bands shown in the numbers in the hrCN-PAGE **(A)**. The original photograph of the hrCN-PAGE **(A)** and source data of **(B–D)** were included in **Supplementary Data 5**. Absorbance spectra were normalized at 679.5 nm. a.u.: arbitrary unit. The CN-PAGE and data of **(B–D)** were representative of two independent experiments.

**Figure 3:**
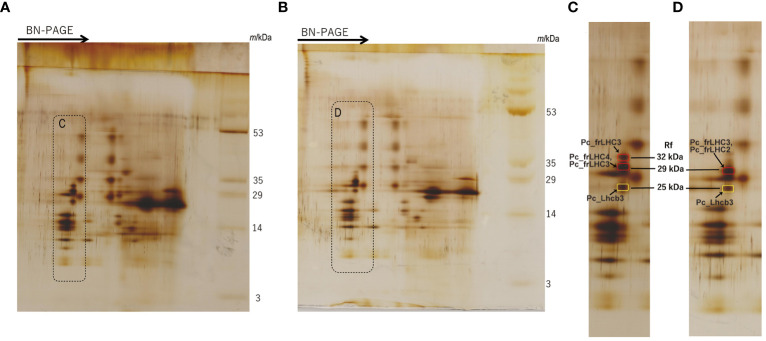
2D-SDS-PAGE of thylakoids isolated from the culture strain incubated under red LED light for 2 weeks **(A)** and harvested cells from the Antarctic habitat **(B)**. Protein complexes in solubilized thylakoid samples were separated by Blue Native (BN) PAGE before 2D-SDS-PAGE. The arrows in **(A, B)** indicate the migration direction in BN-PAGE. **(C, D)** are magnified images of the dotted areas shown in **(A, B)**, respectively. Marked peptide bands in **(C, D)** indicate the MS samples. The original photographs of 2D-SDS-PAGE were included in **Supplementary Data 5**. The CN-PAGE of **(A, B)** were representative of two independent experiments. MS analysis was performed two times for one sample.

The separated greenbands by hrCN-PAGE were cut from the gels and subjected to absorption and fluorescence spectral measurements. We found a prominent far-red absorbing band in bands 3 and 8 isolated from the Antarctic sample and from culture under red LED, respectively (**Figure 2**). An individual sample lane of hrCN-PAGE was cut out and applied for subsequent SDS-PAGE after denaturation to characterize the polypeptide composition of each protein complex (**Figure 3**). Bands 3 and 8 of hrCN-PAGE, which were anticipated to contain Pc-frLHC, showed protein bands around 30 kDa. Specifically, the protein band at 29-kDa (**Figures 3C, D**) was presumed to be a subunit of Pc-frLHC because of the molecular weight deduced from cryo-EM analysis (Kosugi et al., 2023). Together with the 29-kDa protein, polypeptides of 32- (**Figure 3D**) and 25-kDa (**Figure 3C**) were detected in band 8 from the cultured sample under red light. The 32-kDa polypeptide was not clearly detected in band 3 from the sample obtained in Antarctica (**Figure 3**).

**Figure 4:**
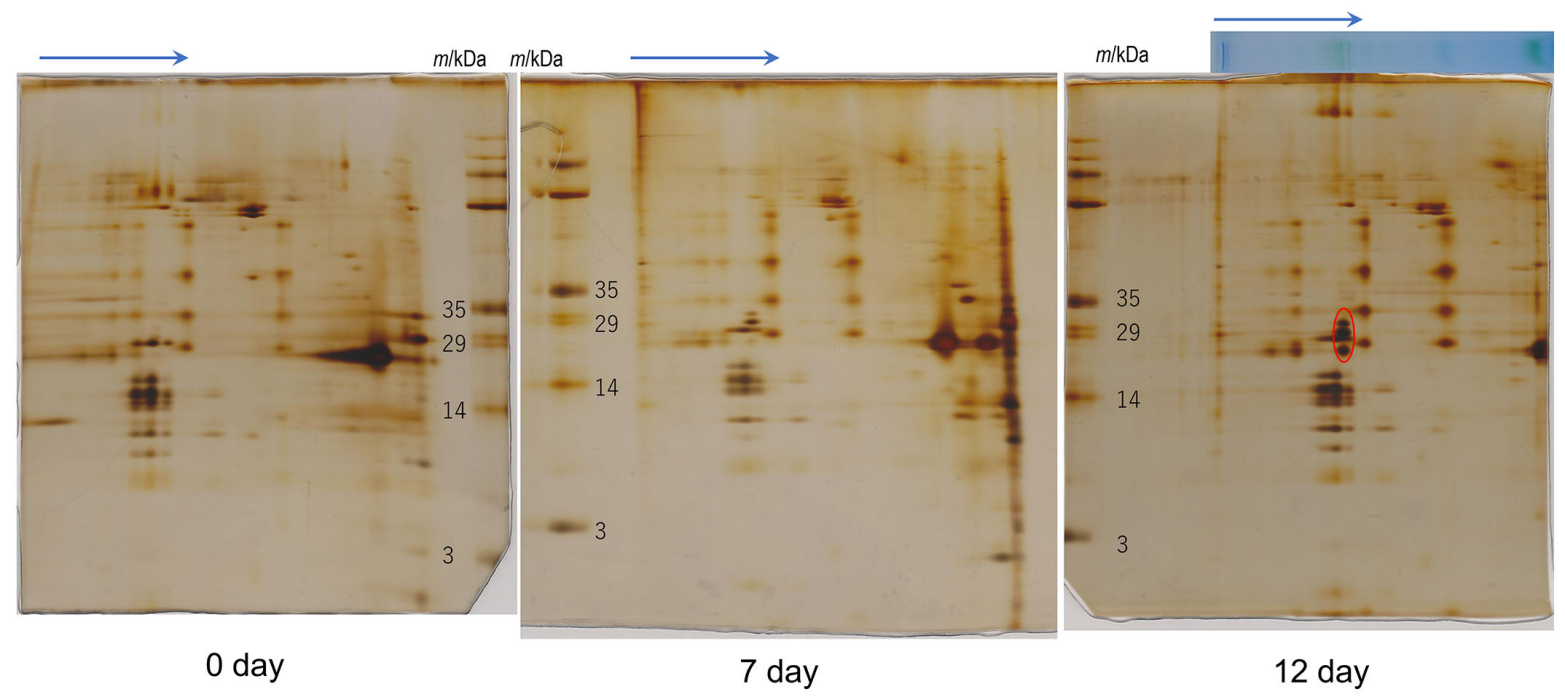
2D-SDS-PAGE of thylakoids isolated from the culture strain. Protein complexes in solubilized thylakoid samples were separated by Blue Native (BN) PAGE before 2D-SDS-PAGE. Arrows indicate the migration direction in BN-PAGE. The day number under each gel (0, 7 and 12 days) indicates the incubation periods (days) under the red LED light after cultivation under fluorescence light about 2 months. The “0 day” sample was used for analysis without red-light incubation. The red circle in the 12-day PAGE gel shows proteins constituting protein complexes induced under red LED light. The original photographs of 2D-SDS-PAGE were included in **Supplementary Data 5**. Number of repetitions was one.

For transcriptome analysis, Pc-frLHC accumulation in the cultured cells was confirmed by 2D-SDS-PAGE in advance. The protein accumulation pattern analyzed by 2D-SDS-PAGE of thylakoids from cultured cells incubated under different light conditions is shown in **Figure 4**. The three protein bands of 32-, 29-, and 25-kDa shown in **Figure 3C** were not detected before starting with red LED illumination (0 day) but gradually accumulated under red LED illumination for 7 and 12 days (**Figure 4**).

### 3.3 Assembly, gene prediction, and genome completeness

Next-generation sequencing data of the *P. crispa* genome indicated an estimated nuclear genome size of 92.2 Mbp, distributed in 1,045 scaffolds with an N50 of 340 kbp and a GC content of 49.7%. Using BRAKER, we predicted 210,244 nuclear genes. The assembly also includes chloroplast scaffolds predicted by DOGMA. BUSCO analysis indicated that 87.1% of the conserved single-copy genes in Eukaryota_odb10 were in the *P. crispa* nuclear genome.

The total size of the mitochondrial genome was 100,066 bp. It contained 529 ORFs and 234 genes, with a GC content of 29.2%. The total sequence length of the chloroplast genome was 211,011 bp in one scaffold, with a GC content of 28.7%. It contained 681 ORFs and 297 genes. The Blast2GO annotated genes on the nuclear and organelle genomes are shown in **Supplementary Data 2**. The genes encoding subunits for PSI and PSII, common in the chloroplast genomes of green algae, were also present in the chloroplast genome of *P. crispa*.

### 3.4 LHC genes in *P. crispa*

Four genes, pcri_g8716 (*Pc_frLHC1*), pcri_g8718 (*Pc_frLHC2*), pcri_g8720 (*Pc_frLHC3*), and pcri_g8045 (*Pc_frLHC4*), were considered to encode Pc-frLHC. The transcripts of these genes showed high homology to the amino acid sequence designated as Pc-frLHC in the transcriptome data (PRJNA329112) used for the 3D structural model (PDB:8HW1) (Kosugi et al., 2023). Twenty-three genes were annotated to LHCs, including those for LHCII, LHCI, and Pc-frLHC, by Blast2GO analysis; however, *LHCSR* genes expressed by high light stress in green algae were not present. We performed an additional search of LHC genes among the nonannotated genes of the draft genome by local blast search using the amino acid sequence of LHCSR of *C. reinhardtii* as a reference. This way, we identified three additional potential Chl *a/b* binding proteins, pcri_g7307, pcri_g1166, and pcri_g3292. Finally, 27 sequences of LHC proteins were obtained from the 26 putative LHC genes in *P. crispa* (**Table 1**)

### 3.5 Identification of Pc-frLHC subunits

The MS database of *P. crispa* for protein identification was constructed from the deduced protein sequences in this study. MS analysis was performed for the spots of these polypeptides separated by 2D-SDS-PAGE (**Figures 3C, D**, and **Supplementary Data 3**). Using MS data, the 29-kDa polypeptide from the culture under red LED light (**Figure 3C**) was assigned to Pc_frLHC4 and Pc_frLHC3, and the 29-kDa polypeptide from the sample harvested from Antarctica (**Figure 3D**) was assigned to Pc_frLHC2 and Pc_frLHC3. Pc_frLHC1 was excluded from these candidate(s) because its unique amino acid stretches were not detected in the MS analysis (**Supplementary Data 3**). The 32-kDa polypeptide detected only in the red light cultured sample (**Figure 3C**) was assigned to Pc_frLHC3. The 25-kDa polypeptides detected in the cultured and Antarctic samples (**Figure 3C, D**) were assigned to one of the major LHCII, pcri_g2502.t1.p1 (Pc_Lhcb3).

### 3.6 RNA-seq analysis in the accumulation process of Pc-frLHC

**Figure 5:**
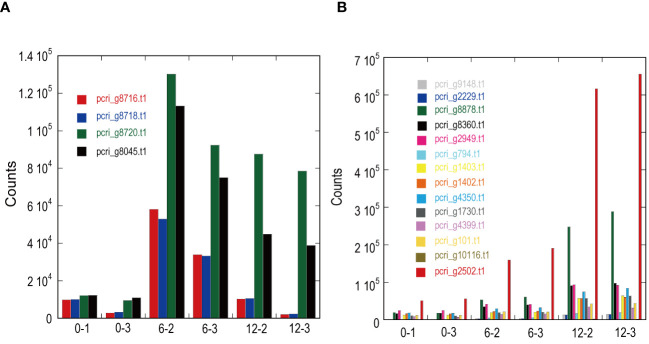
Expression patterns of light-harvesting antenna and photosystems in ExGroup1_i6-d12 **(A)** and ExGroup1_i6-i12 **(B)**. The x-axis indicates the sample IDs used for RNA-seq analysis. 0–x, 6–x, and 12–x indicate the incubation period (days) under red LED light shifted from fluorescence light. The y-axis shows the gene counts with StringTie in RNA-seq analysis.

**Figure 6:**
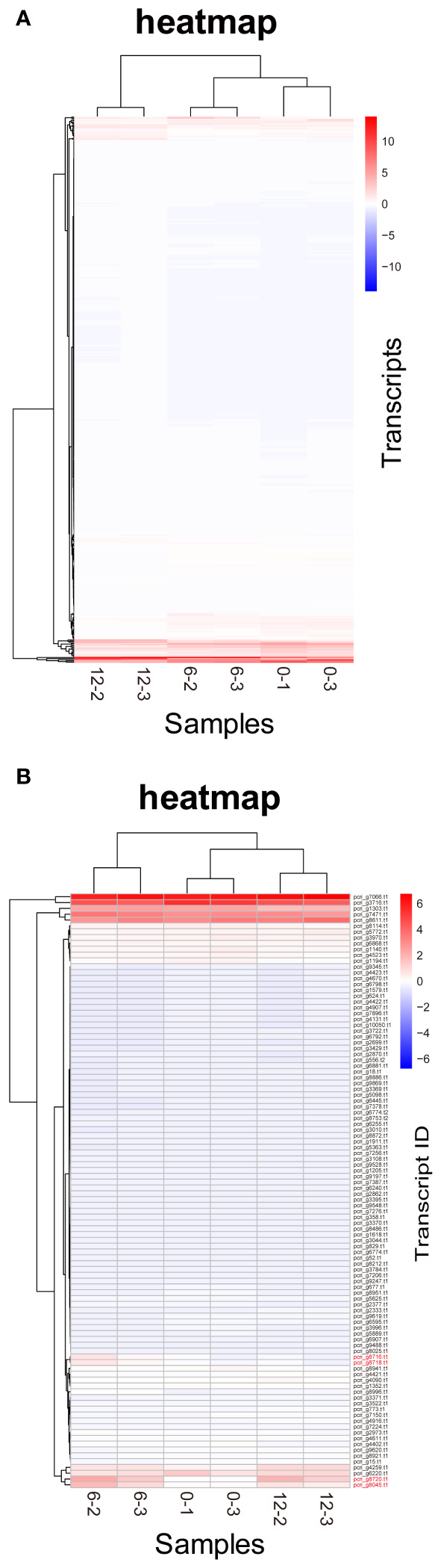
Heatmaps of *P. crispa* transcripts. **(A)** Heatmap of transcripts of 469 genes with increased expression in the first 6 days. **(B)** Heatmap of transcripts of 102 genes annotated in Blast2GO showing an expression pattern similar to that of Pc-frLHC transcripts. Transcript IDs of genes coding Pc-frLHC are shown in red. The values of the matrix are normalized by columns.

DE analysis of the samples under red LED illumination was performed to identify genes coexpressed with Pc-frLHC. We established three conditions: incubation under red LED light at 0, 6, and 12 days. Two transcriptome datasets were prepared for each condition, as described in the Methods section. The expression levels of the transcripts of predicted Pc-frLHC genes (*Pc_frLHC1~4*) increased approximately 10-fold in 6 days and largely or slightly decreased by day 12 (**Figure 5A**). The genes coexpressed with *Pc-frLHC* were identified as follows. All transcripts were rearranged according to the number of log(FC) of DE_0–6; transcripts with log(FC) values <0.5 and FDR values > 0.01 were eliminated. In addition, five genes that exhibited high log(FC) values in DE_0-6 but one of two RNA-seq counts close to zero were eliminated because their evaluation was considered unreliable. At this point, 464 transcripts, including four transcripts of the *Pc-frLHC* genes, remained. The expression patterns are shown as a heatmap in **Figure 6A**. In the next step, we categorized these transcripts into two groups, depending on whether the log(FC) DE_6-12 <0. A total of 295 and 169 transcripts were categorized into the first group (ExGroup1_i6-d12), in which the expression level decreased after day 6, and the second group (ExGroup2_i6-i12), in which the opposite occurred, respectively. It was expected the transcripts of genes coexpressed with *Pc-frLHC* would be categorized in ExGroup1_i6-d12 because of their similar expression profile to *Pc-frLHC*. Of the 295 transcripts in ExGroup1_i6-d12, 102 were annotated through Blast2GO analysis, but the functions of 60% of the remaining transcripts were not assigned. **Figure 6B** shows the heatmap of the annotated 102 transcripts.

**Figure 7:**
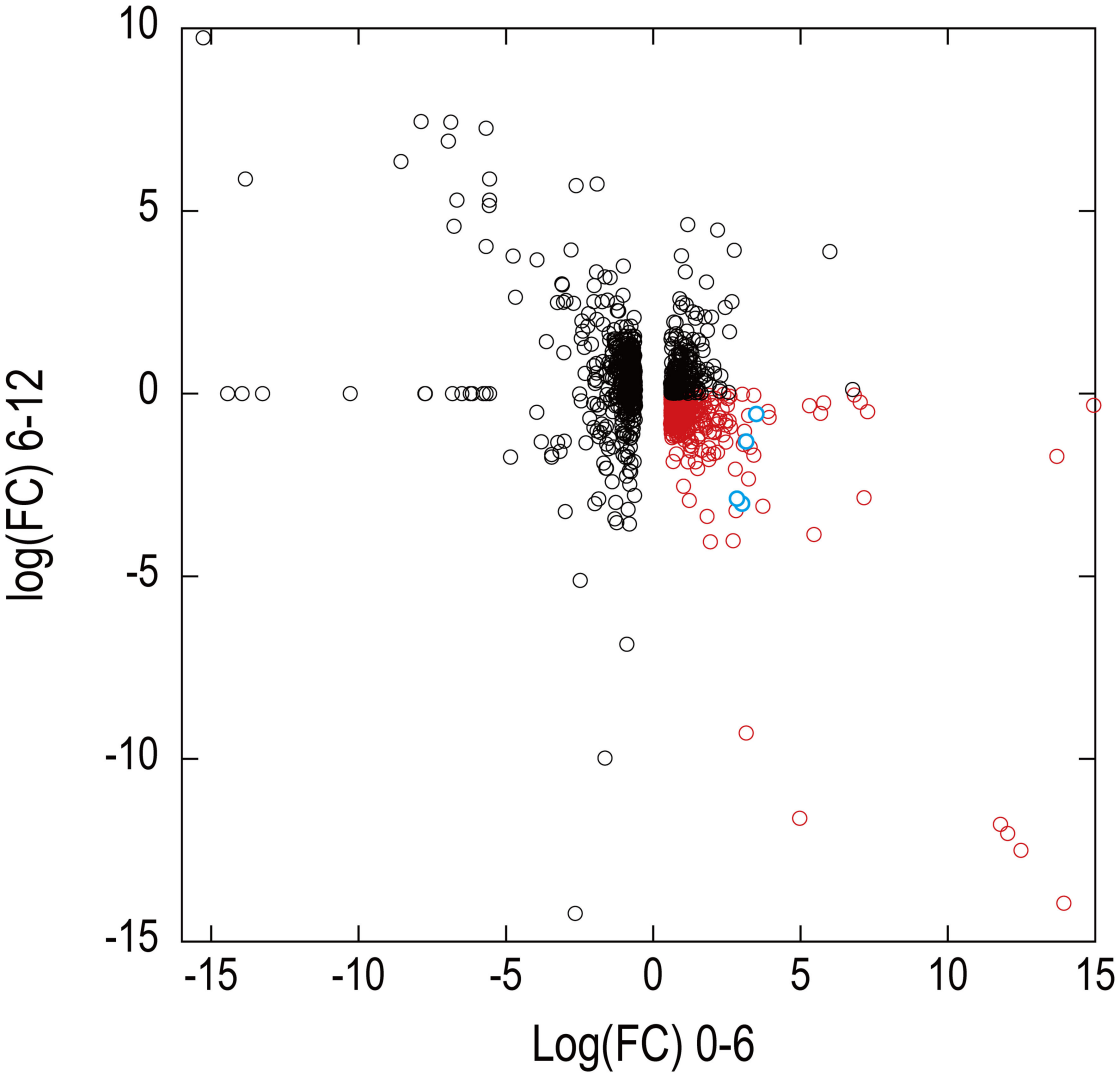
Expression patterns of transcripts identified in the draft genome in RNA-seq analysis. The x-axis and y-axes show the logarithm of fold changes (FC) in the differential gene expression analysis at 0–6 days and 6–12 days, respectively. A total of 9,428 transcripts showing an FDR value >0.01 in the differential expression analysis at 0–6 days were eliminated from the display. Four predicted transcripts of Pc-frLHC genes are shown as blue circles. Red symbols are transcripts of potential genes of coexpression with Pc-frLHC.

**Figure 7** shows the relationship between the log(FC)s of ExGroup1_i6-d12 and ExGroup1_i6-i12. The candidates for the coexpressed genes with *Pc-frLHC* genes and the extraction process are shown in **Supplementary Data 4**. These candidates include genes coding for six transcription factors, four kinases, one phosphatase, five proteins with DNA/RNA binding domains, one calcyclin-binding protein, one calcium sensing receptor, three chaperones, four heat shock proteins, one phototropin, and other metabolic-related proteins. pcri_g8753.t2, encoding “DEAD-box ATP-dependent RNA helicase 35,” showed the highest expression ratio in the first six days. “Niscatine,” pcri_g9345.t1, showed the second highest log(FC) value (5.46) in DE_0-6, but this result was not reproducible because the read counts of the RNA-seq data largely differed between samples 6-2 and 6-3. Interestingly, these transcripts exhibiting high log(FC) values showed limited correlation with *Pc-frLHC* expression in the heatmap as the expression level was quite low compared with that of Pc-frLHC. According to the heatmap, “ATP-binding cassette transporter” and “CCT-domain-containing protein” showed the highest correlation with pcri_g8720.t1 and pcri_g8745.t1 expression. None of the transcripts of the genes encoding for PSI, PSII, or LHC other than *Pc-frLHC* were included in ExGroup1, but transcripts from the genes for LHCII (pcri_g3381.t1, pcri_g5581.t1, pcri_g8360.t1, pcri_g8878.t1, pcri_g2229.t1, pcri_g9148.t1, pcri_g2949.t1, and pcri_g2502.t1), LHCI (pcri_g794.t1, pcri_g1402.t1, and pcri_g1403.t1), and some subunits of the photosystem reaction centers, including PsbQ (pcri_g4399.t1), PsaG (pcri_g101.t1), PsaO (pcri_g4350.t1), and PsbP (pcri_g1730.t1), were present in ExGroup2_i6-i12, whose expression constantly increased over the 12 days of incubation (**Figure 5B**). The expression patterns of chloroplast-encoded genes, such as reaction center proteins of PSII/PSI, were not analyzed because such mRNAs lack poly A-tails and could not be collected.

### 3.7 Molecular phylogenetic analysis of the far-red LHC in *P. crispa*

**Figure 8:**
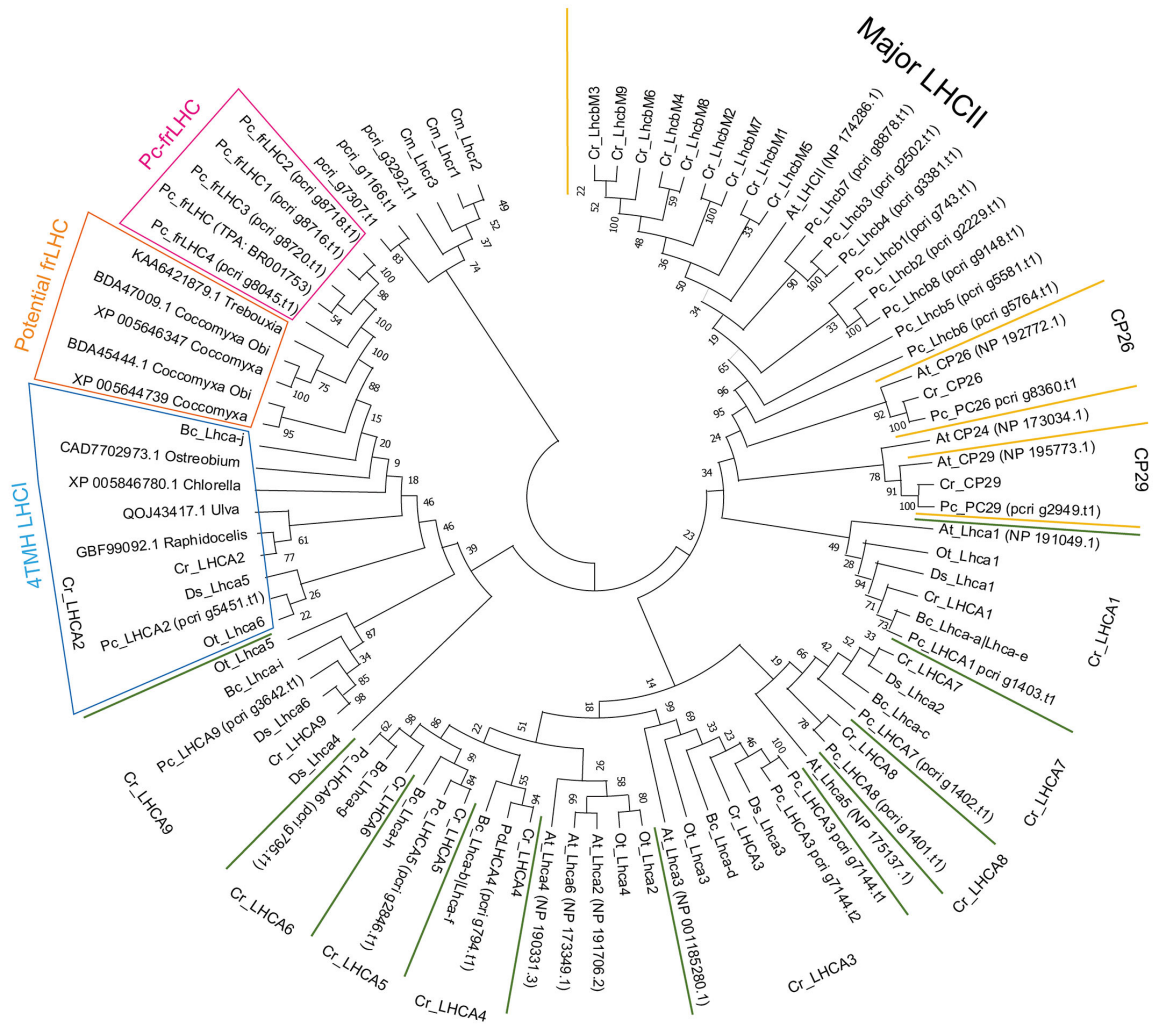
Phylogenetic tree of the LHC. Phylogenetic analysis of LHCs found in the draft genome of *P. crispa* using other green algal LHCs using the maximum likelihood method and the JTT matrix-based model. The amino acid sequences used for the analysis are shown in **Supplementary Data 1**. Cm, *Cyanidioschyzon merolae* (red alga); Cr, *Chlamydomonas reinhardtii*; Bc, *Bryopsis corticulans*; Ds, *Dunaliella salina*; Ot, *Ostreococcus tauri*; Pc, *Prasiola crispa* (green algae); At, *Arabidopsis thaliana* (plant). *Asterisks* on gene names indicate green algae-specific LHCI.

The constructed phylogenetic tree shows that the Pc-frLHC proteins of *P. crispa* form an independent clade of the LHCA clade for LHCI (**Figure 8**). Five functionally unknown LHCs in *Coccomyxa subellipsoidea* C-169 (SAMN02744078), *Coccomyxa* sp. Obi (SAMD00209879), and *Trebouxia* sp. A1-2 (SAMN12598962) form a single clade located between those for *Pc-frLHC* and a noncanonical LHCI, which carries four TMH instead of three. These five functionally unknown LHCs were tentatively classified as potential far-red (fr) LHCs. *C. reinhardtii* has nine *Lhca* genes, and the 10 LHCA proteins encoded by these *Lhca* genes were identified as subunits of the PSI–LHCI supercomplex (Stauber et al., 2003; Tokutsu et al., 2004; Su et al., 2019; Suga et al., 2019). The 10 LHC proteins of *P. crispa* were distributed among the nine clades of Lhca of green algae. The phylogenetic analysis suggests that *P. crispa* has nine *Lhca* genes synonymous with those (*LHCA1-9*) in *C. reinhardtii*.

Eight *P. crispa* LHCs are present in the LHCII clade (**Figure 8**). pcri_g8360.t1.p1 and pcri_g2949.t1.p1 belong to the CP26 and CP29 clades, respectively. pcri_g1166.t1.p1 and pcri_g7307.t1.p1 are closely related to *LHCSR1*/*3* of *C. reinhardtii*, and pcri_g3292.t1.p1 is located between *Lhcrs* of red alga and *LHCSRs* in the tree. **Table 1** shows the proposed function of *P. crispa* LHCs based on phylogenetic analysis.

**Figure 9:**
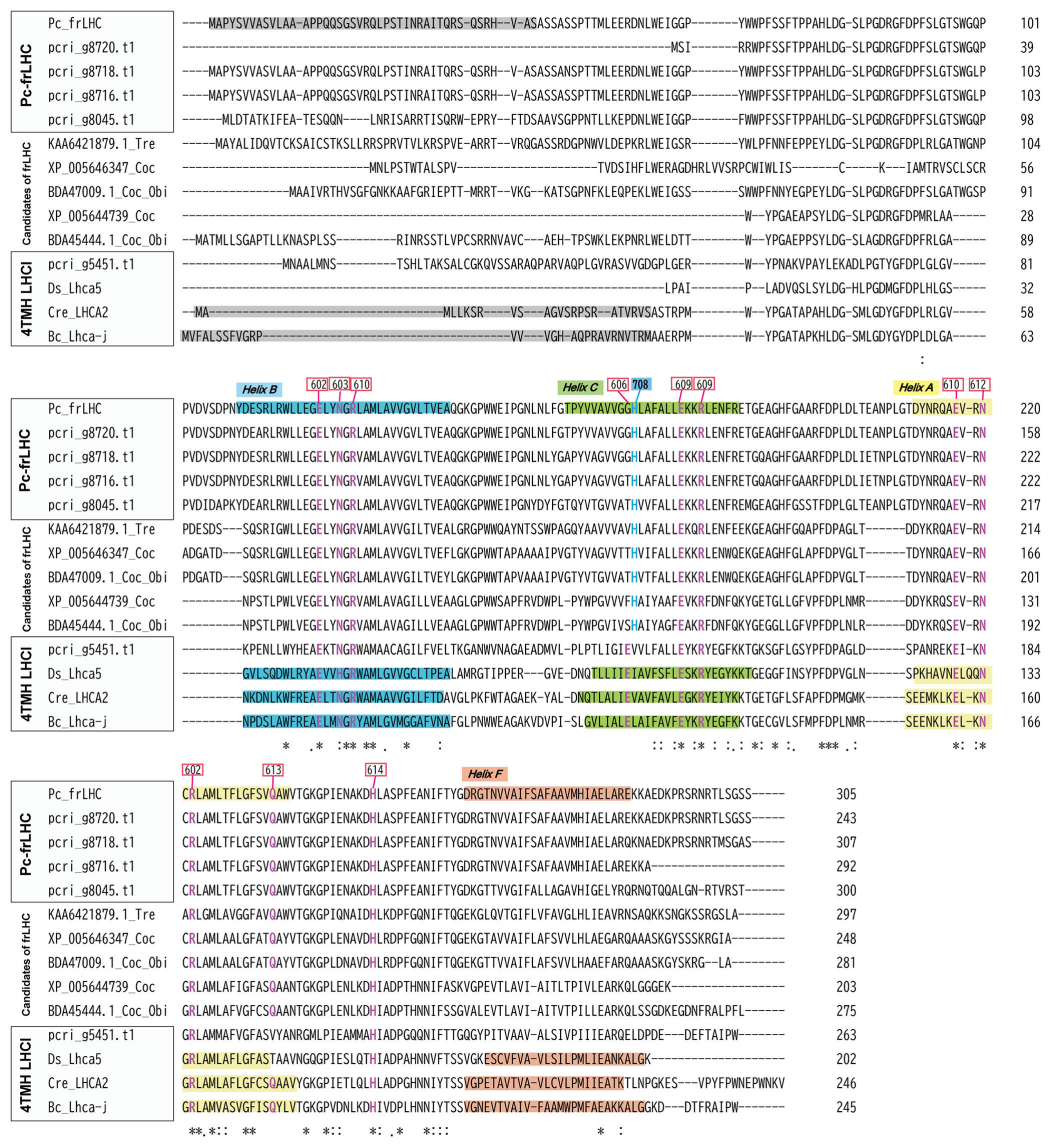
Sequence alignment of Pc-frLHC from *P. crispa* and closely related LHCs. The deduced amino acid sequences of LHC in the clades of Pc-frLHC, candidates of far-red absorbing LHC (frLHC), and 4-TMH LHCI aligned with Clustal Omega (EMBL-EBI). The regions coding for transmembrane helices are shaded in different colors. The signal peptides are shaded. Chlorophyll-binding residues (following the nomenclature of Liu et al.) are shown in red. The signal peptides and transmembrane helices were predicted from 3D structures registered in the PDB (Cr_Lhca2; 6JO5, 6IJO, Bc_LhcaJ; 6IGZ, Ds_Lhca5; 6SL5) and from the results of secondary structural prediction using Jpred 448 and TargetP-2.049. The Chl708 binding sites are shown in blue.

**Figure 9** shows the alignment of the deduced amino acid sequences of the five Pc-frLHCs, the five potential frLHCs of *Coccomyxa* sp. or *Trebouxia* sp., and the 4TMH LHCIs. Histidine ligands for Chl708 in Pc-frLHC were present in the five potential frLHCs but not in 4TMH LHCIs. The glutamic acid ligands for Chl606 in 4TMH LHCI were not present in Pc-frLHC or the potential frLHCs.

## 4 Discussion

### 4.1 Evolutional process of Pc-frLHC

Phylogenetic analysis of the LHC sequences obtained in this study revealed that Pc-frLHC is closely related to LHCI, with four TMH (4TMH LHCI) (**Figure 8**). Cr_LHCA2, Ds_Lhca5, and Bc_LhcaJ are in the relationship of synonyms from the structural analysis of PSI-LHCI complexes (Qin et al., 2019; Su et al., 2019; Suga et al., 2019; Caspy et al., 2020). The fourth transmembrane helix, *helix-F*, plays an important role in the association with PSI by hydrophobic interaction with a helix in PsaI, which is a peripheral subunit of PSI (Su et al., 2019; Suga et al., 2019). The early-branching green algal species *Ostreococcus tauri* also has a 4-TMH LHCI Ot_Lhca6 (PDB:7YCA_6). This was identified by the 3D structural model of the PSI– LHCI–LHCP supercomplex, and the binding position to PSI was the same as that of Cr_LHCA2 (Ishii et al., 2023). The 4-TMH LHCI in green algae is thus considered as an early evolutionary acquisition within the green lineage. It is likely to have coevolved with a partner protein in the heterodimer, such as LHCA9 in *C. reinhardtii*.

Pc-frLHC exhibits high homology to 4-TMH LHCI, but there is an important substitution in the amino acid residue that binds to Chl708 in Pc-frLHC (Kosugi et al., 2023). This substitution may have enabled the formation of far-red absorbing Chls. The same amino acid substitution was found in the potential frLHCs in *Trebouxiaceae*, *Coccomyxa subellipsoidea* C-169, *Coccomyxa* sp. Obi, and *Trebouxia* sp. A1–2 (**Figure 9**). *Trebouxia* sp. A1-2 is a photobiont of the lichen *Lasallia pustulata* (PRJNA464168). Although the photosynthetic abilities of these species using far-red light are not clear, they may have such a potential given that they possess the ortholog *Pc-frLHC*.

Aquatic organisms rarely encounter environments with a high irradiance of far-red light compared with visible light as far-red light is preferentially absorbed by water and its intensity rapidly decreases with water depth, making visible light more prevalent in aquatic environments (Kirk, 2010). Thus, it would be interesting to study when and why frLHC evolved from the normal 4-TMH LHCI in the green lineage as photosynthesis using far-red light is beneficial only under terrestrial or shallow water conditions (Hale and Querry, 1973; Stomp et al., 2007). The Trebouxiaceae family comprises many aerial and soil algal species that sometimes inhabit environments with limited visible light (Lemieux et al., 2014; Kosugi et al., 2023). Thus, the development of frLHC was crucial for survival in their habitat. At present, putative frLHCs showing high homology to Pc-frLHC are only found in the Trebouxiaceae family. This suggests that frLHC, which contributes to PSII excitation with far-red light in *P. crispa*, diversified from the 4-TMH LHCI in a green alga during expansion to land after Trebouxiaceae evolution in the green linage. However, the evolutionary process whereby LHCI was adapted to function as an LHC for PSII remains unclear. The increasing genome sequence data accumulated and identification of species possessing far-red LHC may help clarify the evolutionary process of Pc-frLHC.

### 4.2 Different expression pattern of Pc-frLHC genes

In this study, four putative *Pc_frLHC* genes were identified in the draft genome sequence. The deduced amino acid sequences of these proteins, Pc_frLHC1, Pc_frLHC2, and Pc_frLHC3, exhibit high homology. As three of the four genes are located at close positions in the genome, gene duplication(s) likely occurred during evolution of the *Pc_frLHC* genes. Another *Pc_frLHC* gene, *Pc_frLHC4*, shows lower homology than other *Pc-frLHC* genes and is located apart. RNA-seq analysis revealed that all *Pc-frLHC* genes were expressed in cultures incubated under red LED illumination. However, *Pc_frLHC1* and *Pc_frLHC2* were not detected by MS analysis of the red illuminated cells (**Figure 3**). The expression of both *Pc_frLHC1* and *Pc_frLHC2* largely decreased at 6–12 days under red LED illumination, whereas *Pc_frLHC3* and *Pc_frLHC4* continued to be expressed. As a result, only Pc_frLHC3 and Pc_frLHC4 accumulated after long growth periods (**Figure 5**). Interestingly, MS analysis revealed that *Pc_frLHC4* was expressed in the cultured cells but not in the samples from Antarctica (**Figure 3**). Based on these results, two possibilities emerged: 1) *Pc_frLHC4* is specifically expressed under red light but not in the natural habitat, or 2) the cultured strain and Antarctic sample have different *Pc-frLHC* gene sets, with only *Pc_frLHC4* present in the cultured strain. The second hypothesis is plausible because the cultured strain was isolated from sands collected in an area different from that where the Antarctic sample was harvested.

MS analysis revealed that both the 32-kDa and 29-kDa polypeptides in the cultured cells were Pc-frLHC proteins encoded by *Pc_frLHC3* (**Figure 3**, **Supplementary Data 3**). It is possible that the 32-kDa protein is a modified version, by phosphorylation for instance, of the 29-kDa protein, or it could be a precursor of the 29-kDa protein with a transit peptide. Protein phosphorylation is usually observed in LHC on the thylakoid membrane for function regulation (Rochaix, 2007). The25-kDa protein separated in hrCN-PAGE was assigned to one of the major LHCIIs, Pc_Lhcb3, by MS analysis (**Figure 3**). Pc_Lhcb3 was coexpressed with *Pc-frLHC*, but its basic expression level was high and constantly increased under illumination with red LED light (**Figure 5B**). Therefore, Pc_Lhcb3 may be associated with both Pc-frLHC and PSII in thylakoid membranes, and some Pc_Lhcb3s may have been isolated with Pc-frLHC.

DE analysis revealed that the expression of *Pc-frLHC* genes increased at the onset of red LED illumination and reached a maximum after 6 days (**Figure 5A**). This expression pattern can help select the coexpressing genes of *Pc-frLHC* from those of other LHCs whose expression continues to increase over 12 days. The decrease in expression after 6 days suggested some feedback mechanism that controls the expression of those genes.

### 4.3 Function of the coexpression factors

The gene of pcri_g8753.t2 was annotated as “DEAD-box ATP-dependent RNA helicase 35.” Since it exhibited the highest FC value in DE0_6, this gene might act as a specific translation factor for Pc-frLHC, given that it was not transcribed before illumination under red LED light (**Supplementary Data 4**). DEAD-box proteins constitute a protein superfamily distributed from bacteria to mammals. Various activities have been confirmed for these proteins, including not only RNA helicase activity but also stabilization of mRNA, splicing of mRNA, and ribosome assembly in the processes of RNA transcription and translation (Linder and Jankowsky, 2011).

The “CCT (CO, CO-like, and TOC1) -domain-containing protein” encoded by pcri_g4259.t1, which showed a high correlation of expression with Pc-frLHC genes, *Pc_frLHC3* and *Pc_frLHC4*, in the heatmap (**Figure 6B**), is a transcriptional factor of responses to environmental signals and has roles in measuring photoperiod, setting circadian rhythms, and other functions in plants (Shen et al., 2020). Microalgae also have “CCT-domain containing proteins” but information on the function of these proteins is limited. Serrano et al. reported that CCT-domain-containing proteins control cell division by creating a circadian rhythm in *C. reinhardtii* (Serrano et al., 2009). Interestingly, some genes related to blue light receptors, phototropin, and cryptochrome, were identified as genes coexpressed with *Pc-frLHC*. Cryptochrome is a flavin protein that acts as a blue light receptor and involved in triggering many light-induced reactions such as circadian rhythm and flowering in plants (Lin, 2002; Kottke et al., 2017). Phototropin harbors the light, oxygen, or voltage domain(s) (**Supplementary Data 4**). Therefore, it is possible that Pc-frLHC expression is negatively regulated by these blue light photoreceptor(s). This hypothesis is supported by the observation that Pc-frLHC was induced by red LED light but not under white fluorescent light. These results are also consistent with those by Kosugi et al. (2023), indicating that Pc-frLHC is expressed in the lower layer of *P. crispa* colonies, where blue light is extremely limited in the Antarctic habitat (Kosugi et al., 2023).

Heat shock proteins were induced to some extent concomitant to *Pc-frLHC* expression. Heat shock proteins are not only induced by heat or other stresses but are also induced to assist protein folding and transport to organelles. HSP70 participates in protein folding and transfer to organelles as a chaperone. Four transcripts, pcri_g7471.t1, pcri_g7066.t1, pcri_g8114.t1, and pcri_g7276.t1, annotated as HSP70, are likely involved in folding and stabilization of Pc-frLHC for transfer to the chloroplast. HSP70 is also a cold response chaperone in algae, plants, insects, and animals (Jiang et al., 2012; Maikova et al., 2016; Storey and Storey, 2023). These chaperones and the “DEAD-box ATP-dependent RNA helicase 35” coded by pcri_g8753 may play a crucial role in increasing the translational efficiency and stable synthesis of Pc-frLHC in the low-temperature environment of Antarctica.

## 5 Conclusion

Pc-frLHC has four transmembrane helices homologous to LHCA2 in *C. reinhardtii*. Its expression is regulated by light quality and is independent of other LHCs. *P. crispa* and some green algal species in the Trebouxiaceae family may possess far-red absorbing LHCs and be capable of performing photosynthesis using far-red light. In the future analysis, revealing the molecular mechanism to regulate expression of Pc-frLHC and other LHCs by using the draft genome data is a critical research subject for understanding adaptation and evolution process in far-red light abundant environments on the earth.

## Data availability statement

The datasets presented in this study can be found in online repositories. The names of the repository/repositories and accession number(s) can be found in the article/**Supplementary material**.

## Author contributions

MK: Conceptualization, Data curation, Formal Analysis, Funding acquisition, Investigation, Methodology, Project administration, Resources, Supervision, Validation, Visualization, Writing – original draft, Writing – review & editing. SO: Data curation, Investigation, Methodology, Resources, Supervision, Visualization, Writing – original draft. KH: Data curation, Formal Analysis, Investigation, Methodology, Resources, Supervision, Visualization, Writing – original draft. AT: Data curation, Formal Analysis, Investigation, Methodology, Software, Supervision, Visualization, Writing – original draft. HN: Data curation, Formal Analysis, Investigation, Methodology, Supervision, Visualization, Writing – original draft. S-IO: Data curation, Formal Analysis, Investigation, Methodology, Visualization, Writing – original draft. YT: Resources, Writing – review & editing, Methodology. YK: Funding acquisition, Methodology, Writing – review & editing. SK: Methodology, Resources, Writing – review & editing. HK: Conceptualization, Methodology, Resources, Writing – review & editing. JM: Funding acquisition, Resources, Writing – review & editing.
