# Supplementary material for: Characterization of the far-red light absorbing light-harvesting chlorophyll a/b binding complex, a derivative of the distinctive Lhca gene family in green algae
Source: Front Plant Sci. 2024 Jun 10;15:1409116. doi: 10.3389/fpls.2024.1409116 (PMC11194369; doi:10.3389/fpls.2024.1409116)
Supplement: Supplementary file 1 [file DataSheet_1.docx]

Supplementary Material

**Characterization of the far-red light absorbing light-harvesting chlorophyll *a*/*b* binding complex, a derivative of the distinctive Lhca gene family in green algae**

Makiko Kosugi*, Shuji Ohtani, Kojiro Hara, Atsushi Toyoda, Hiroyo Nishide, Shin-Ichiro Ozawa, Yuichiro Takahashi, Yasuhiro Kashino, Sakae Kudoh, Hiroyuki Koike and Jun Minagawa

*Correspondence: M. Kosugi (mkosugi@nibb.ac.jp)

**1 Supplementary Data**

**Supplementary Data 1.** Amino acid sequences for Phylogenetic tree (external)

**Supplementary Data 2.** Annotation of genes(external)

**Supplementary Data 3.** MS analysis(external)

**Supplementary Data 4.** RNA-seq analysis(external)

**Supplementary Data 5.** SapplementaryData5_Source Data (external)

**2 Supplementary Figure**


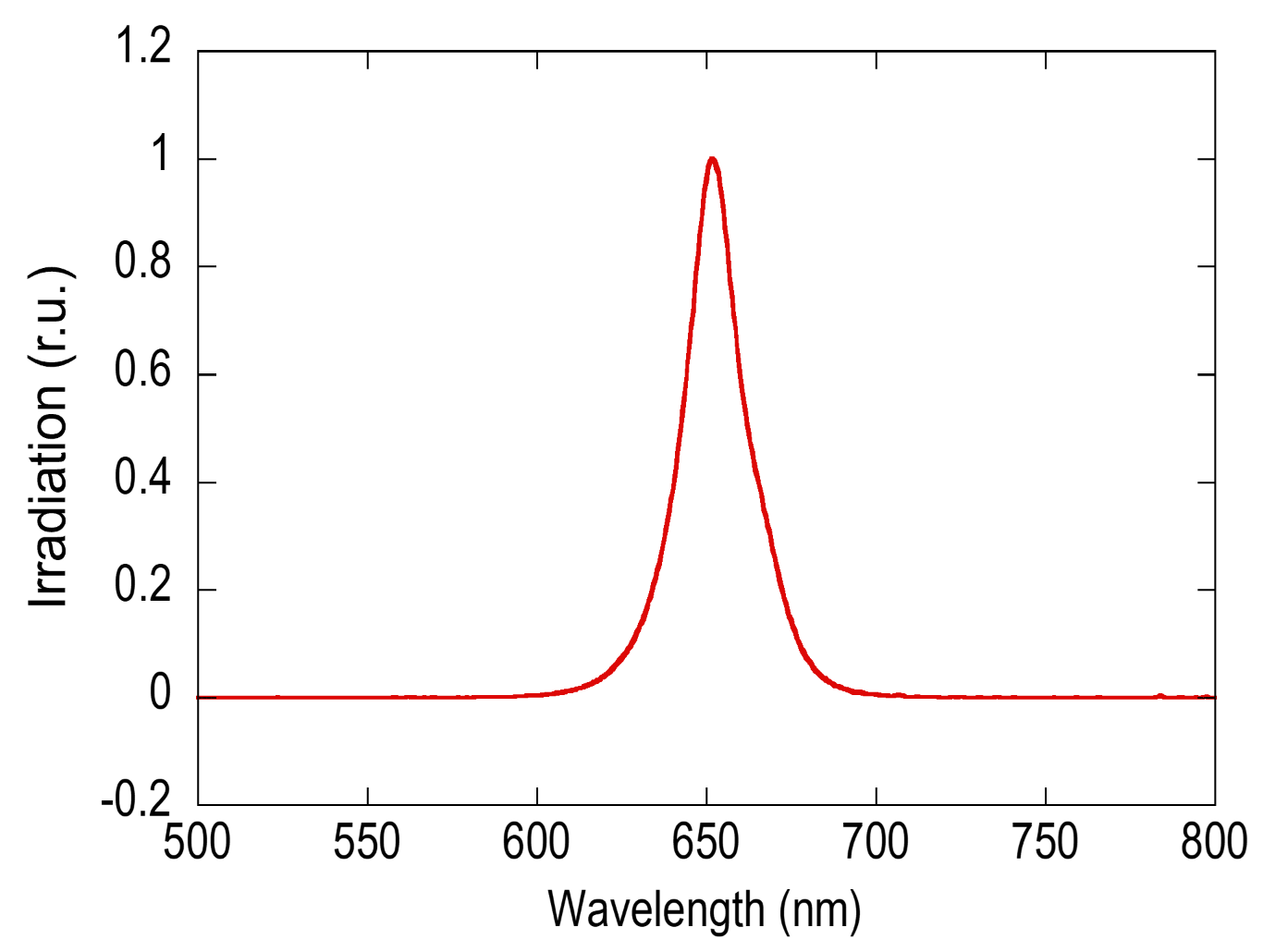


**Supplementary Figure 1**. The spectrum of the red LED light used to induce Pc-frLHC that was measured by a spectrometer, Black-Comet (StellarNet, Tampa, FL, USA). The irradiation (W/m^2^) was normalized at the peak wavelength. r.u.: relative unit.

**3 Supplementary Table**

**Supplementary Table 1**.

The quality data of total RNA samples. A260 and A280 are absorbance at 260 and 280 nm, respectively. The samples used for RNA-seq analysis are shown in bold characters.

| **Sample** | **ng/uL** | **A260** | **A280** | **A260/A280** | **Total (ug)** |
| --- | --- | --- | --- | --- | --- |
| **0-1** | **572.1** | **14.30** | **6.80** | **2.10** | **17.2** |
| 0-2 | 381.5 | 9.54 | 4.34 | 2.20 | 11.4 |
| **0-3** | **551.8** | **13.80** | **6.49** | **2.13** | **16.6** |
| 6-1 | 2397.5 | 59.94 | 27.57 | 2.17 | 71.9 |
| **6-2** | **775.6** | **19.39** | **8.91** | **2.18** | **23.3** |
| **6-3** | **782.6** | **19.57** | **9.06** | **2.16** | **23.5** |
| 12-1 | 2190.2 | 54.75 | 24.95 | 2.19 | 65.7 |
| **12-2** | **981.9** | **24.55** | **11.13** | **2.21** | **29.5** |
| **12-3** | **995.3** | **24.88** | **11.31** | **2.20** | **29.9** |
